# Smart Home Technology Integration in Home Modification Programs Serving Older Adults: Focus Group Study With Program Grantees

**DOI:** 10.2196/89729

**Published:** 2026-07-16

**Authors:** Haomin Hu, Moriah Clarke, Meredith Hughes, Palash Sharma, Jack Fried, Portia Singh, Pamela Toto

**Affiliations:** 1 Department of Health Information Management, University of Pittsburgh, 3396 Fifth Ave, Room 4178, Pittsburgh, PA, 15213, United States, 1 412 503 3886; 2 Department of Occupational Therapy, University of Pittsburgh, Pittsburgh, PA, United States; 3 Department of Health Policy and Management, University of Pittsburgh, Pittsburgh, PA, United States; 4 Health Policy Institute, University of Pittsburgh, Pittsburgh, PA, United States; 5 Department of Rehabilitation Science and Technology, University of Pittsburgh, Pittsburgh, PA, United States

**Keywords:** smart home technology, aging in place, home modification program, internet of things, technology adoption

## Abstract

**Background:**

As people age over the coming decades, demand for in-home support and other interventions, such as home modifications, to help older adults age in place successfully, is also expected to rise. Smart home technologies have the potential to enhance aging in place by complementing traditional home modifications; however, adoption within federally funded home modification programs remains limited.

**Objective:**

This study explored grantees’ perspectives on the Older Adults Home Modification Program (OAHMP) to understand current practices, perceived benefits, barriers, and strategies for integrating smart home technologies into home modification services.

**Methods:**

An exploratory qualitative study was conducted with staff, occupational therapists, and home builders from 3 OAHMP grantee organizations. A 1.5-hour virtual focus group was held and thematically analyzed using a deductive approach grounded in the discussion agenda.

**Results:**

A total of 11 participants reported early adoption of select smart devices—most commonly smart speakers, doorbell cameras, motion-activated lights, and smart plugs—to enhance home safety, communication, and independence. Barriers to broader implementation emerged at three levels: (1) older adults’ digital literacy, privacy concerns, and device maintenance burden; (2) contextual constraints, such as unreliable internet in rural areas; and (3) organizational limitations, including training needs, staffing capacity, and funding challenges. Participants emphasized the importance of progressive adoption, hands-on training, and low-maintenance technology. In addition, partnerships with university educational programs were well established among selected grantees to provide technology training resources for older adults. To inform a reasonable technology selection for home modifications, criteria were proposed across 5 domains: installation, usability, accessibility, sustainability, and security and privacy.

**Conclusions:**

Integrating smart home technologies into home modification programs provides a scalable, cost-effective opportunity to improve aging in place outcomes. Policy support, workforce training, valid selection criteria, and sustainable funding models are needed to promote equitable adoption across OAHMP and similar federally supported programs.

## Introduction

### Background

The share of Americans aged 65 years and older is projected to increase from 17% to 22% by 2040, and the number of Americans aged 85 years and older is projected to double over that same period [1]. The prevalence of disability increases with age, as approximately 14% of adults aged 65 to 74 years and 29% of adults aged 75 years and older have an ambulatory difficulty, defined as a challenge walking or climbing stairs, and approximately 11% of the population aged 75 years and older has a self-care difficulty, defined as a challenge with bathing or dressing [2]. As the population ages over the coming decades, demand for in-home support and other interventions, such as home modifications, to help older adults age in place successfully, is expected to rise.

“Aging in place” refers to maintaining functional independence and social connections while living in the home or community [3]. A national survey reported that 75% of adults aged 50 years and older wish to stay in their own homes as they age, and 73% hope to stay in their communities [4], underscoring the importance of residential environments in supporting daily living and well-being. While remaining at home is desired by the vast majority of older adults, many American homes lack key features that provide the best person-environment “fit” to support aging in place [5]. As of 2019, about 40% of homes were considered aging-ready, with a bedroom and full bathroom on the first floor and a step-free entryway [2]. Today, only 54% of older adults aged 80 years and older reside in single-floor homes with step-free entryways [6]. Correspondingly, demand for in-home support and residential adaptations is expected to continue, placing a greater emphasis on strategies that enable older adults to live safely and independently.

### Home Modifications to Support Aging in Place

One of the most widely recognized approaches to supporting aging in place is home modification. Home modifications are a prominent and essential feature of evidence-based programs, such as Community Aging in Place–Advancing Better Living for Elders (CAPABLE), which seek to reduce falls and improve the safety and independence of older adults aging in place [7]. Home modifications—such as installing grab bars, building ramps, or improving lighting—have been shown to reduce environmental hazards, enhance accessibility, and lower the risk of falls or injury [8]. Evidence indicates that such a solution not only improves safety but also delays institutionalization and reduces health care costs [9]. Despite these benefits, current home modification programs face challenges, including limited funding, a lack of service providers, and difficulties tailoring interventions to the diverse needs of older adults and their caregivers, including considerations of cost and time required to implement home modifications [10-13].

The cost of home modifications varies significantly depending on the nature of the alteration: items such as grab bars typically cost a few hundred dollars to acquire and install, whereas wheelchair ramps or stairlifts can cost several thousand dollars. Nearly one-third of older adult households are cost-burdened, defined as spending at least 30% of their income on housing, utilities, taxes, and insurance [6,14]. To address gaps between the need for and access to home modifications, the Older Adults Home Modification Program (OAHMP), established by the US Department of Housing and Urban Development, focuses on high-impact, low-cost home modification solutions. The OAHMP grant, created in 2021, provides funding to organizations to support low-income older adults in aging in place through home modifications that enhance the safety and accessibility of their home environment [15].

### Smart Home Technologies in Home Modification Programs

Over the past decade, smart home technologies have undergone rapid evolution and become increasingly prevalent in everyday life. Devices such as voice-activated intelligent assistants (eg, Google Assistant, Alexa, and Siri), remote monitoring systems, motion-sensor lighting, and video doorbells are now widely available at relatively low cost. These technologies offer new opportunities to extend the benefits of traditional home modifications by enhancing safety, improving communication and monitoring, and supporting independence [16]. Their potential application in home modification programs is particularly promising, given the growing emphasis on technology-enabled care and prevention [17].

Although some OAHMP grantee organizations have reported integrating products such as automatic door openers, security systems, and fall-detection devices into their home modification installations, most OAHMP and similar home modification programs do not include smart technologies in their menu of environmental modifications [15]. Incorporating additional smart home technologies into the OAHMP could support a range of aging in place goals. For example, smart, motion-sensitive lighting can improve visibility and reduce fall risk in areas such as stairwells and bathrooms. Smart speakers can enable automated control of door locks, lighting, window blinds, and other devices, helping individuals with reduced mobility maintain control over their environment. Smart plugs and appliances, such as coffee pots or stoves, can enable caregivers to remotely support and monitor daily routines. Despite the widespread availability of these technologies and the growing inclusion of home modification interventions, their adoption in home modification programs remains limited [18]. Barriers, including a lack of standardization, concerns about cost and maintenance, concerns about technology acceptance by older adults, and uncertainty regarding training and support, have prevented consistent integration into service delivery [16]. Additionally, incorporating smart technologies into traditional home modification approaches requires a readiness for change by both providers and consumers, which may inhibit the uptake and diffusion of this innovation for aging in place [19,20].

To better shape future program design and policy support, it is essential to understand how practitioners view the opportunities and challenges of integrating smart home technologies. This study aimed to explore OAHMP grantees’ perspectives on current practices, perceived benefits, barriers, and strategies for integrating smart home technologies into home modification services, and to propose criteria to guide technology selection for program implementation.

## Methods

This was an exploratory qualitative study with thematic analysis of focus group feedback.

### Recruitment

On the basis of sample size recommendations for focus groups [21], the research team worked with leadership from 3 OAHMP grantee organizations representing rural, suburban, and national programs to purposively select 9 to 12 (3‐4 per organization) professionals, consisting of home modification contractors, remodeling contractors, Certified Aging-in-Place Specialists (CAPSs), occupational therapists, other assessment professionals, and home modification program administrators or staff. Recruitment information was emailed to the leaders and subsequently forwarded to English-speaking professionals working for the regional organizations. No professionals were excluded on the basis of gender, race, or ethnicity.

### Procedure

A single focus group session was conducted via Zoom and lasted approximately 1.5 hours. An agenda was prepared in advance, with discussion questions organized into three sections: (1) background and ongoing home assessments and modifications; (2) attitudes, current adoption, and barriers related to introducing smart home technology into home modification programs; and (3) future expectations for integrating smart home technology and potential motivators to support adoption (Multimedia Appendix 1). The diffusion of innovations theory, readiness for change, and technology acceptance models were used to guide question content [19,20,22,23]. Two facilitators (HH and MC) from the research team guided the session, alternating leadership across the 3 sections. The primary facilitator in each section posed questions and encouraged participants to share their perspectives, ensuring that all 3 participating organizations responded with comments. While 1 facilitator engaged with participants, the other took detailed notes to capture key points and emerging themes. The entire session was audio-recorded via Zoom to ensure accurate capture of participants’ responses.

### Data Analysis

The audio recording was transcribed using Zoom’s cloud-integrated transcription feature. Data were analyzed using a deductive thematic analysis approach, guided by the topics outlined in the focus group agenda. A 10-theme codebook was developed in advance based on the agenda questions (Table 1). Two independent reviewers (HH and MC) systematically coded the transcript and assigned responses to the predefined themes. Any discrepancies were reviewed by a third, experienced researcher (PT) until an agreement was reached. Beyond categorization, the analytical process involved interpretive synthesis across coded segments to identify patterns and their broader implications within and across themes. Following this process, the initial 10 themes were refined and synthesized by merging overlapping content and eliminating redundancies, yielding the final set of themes used to structure the results.

**Table 1.** Focus group agenda and the theme codebook.

Theme	Subthemes or a priori codes	Typical indicators
Clients and referrals	Primary target population Referral sources and pathways	“Mostly low-income seniors” “Hospital social workers send us referrals”
Assessment practices	Standard checklist or protocol Assessor roles (occupational therapist, contractor, and staff) Data sources (self-report, observation, and performance demonstration) Scope (entire home vs selected areas)	“We observe meal prep tasks” “We skip bedrooms unless requested”
Time and workflow	Typical timeline (assessment to completion) Factors that lengthen or shorten the process	“Funding approval adds 3 weeks”
Common modifications	Frequently delivered changes Driven by funding rules or client priorities	“Grab bars, handrails” “Ramp only if grant covers it”
Current use of smart home technology and assistive technology	Technologies already installed Decision authority (client, assessor, or others) Staff and contractor training Client and caregiver receptiveness Reasons for not using technology (cost, training, and funding compatibility)	“We add voice-activated lights when caregivers ask”
Perceived benefits	Promoting independence and safety Injury prevention and fall reduction Caregiver peace of mind Staff workload reduction and service efficiency	“Sensors alert us before incidents”
Barriers and concerns	Homeowner resistance or stigma Digital literacy and technology anxiety Data privacy and security concerns Installation complexity and maintenance burden Cost and funding incompatibility	“Clients fear being watched” “Wi-Fi is unreliable”
Proposed solutions and facilitators	Training and education strategies Funding or cost-sharing ideas Technical support models	“Partner with a local tech club for setup help”
Implementation pathways	When and where to integrate technology (assessment stage, standard package, or add-on) Impact on daily practice, coordination, and documentation	“Add a ‘smart tech’ checkbox to assessment form”
Organizational capacity and change	Staff training needs New roles or expertise required Workflow restructuring Leadership buy-in External partnerships (technology vendors, universities, and aging services)	“Need a dedicated tech coordinator”

### Ethical Considerations

This study was reviewed and granted an exempt determination by the University of Pittsburgh Institutional Review Board (STUDY24120137). Verbal informed consent was obtained from all participants at the beginning of the focus group session, during which they were informed of the study’s purpose, the voluntary nature of participation, and that the session would be audio recorded.

## Results

### Characteristics of Participants

A total of 11 participants from the 3 OAHMP grantee organizations attended the focus group interview. Organizational roles of the participants are shown in Table 2. Organizational identifiers are not reported at the individual level to protect participant confidentiality, given the small size of the participating organizations and the risk of identification through role-organization combinations.

**Table 2.** Professional roles of the participants (N=11).

Roles	Participant, n (%)
President or chief executive officer	1 (9.1)
Program manager or director	3 (27.3)
Grant administrator	1 (9.1)
Occupational therapist	1 (9.1)
Certified Aging-in-Place Specialist	4 (36.4)
Home repair lead	1 (9.1)

### Theme 1: Client Characteristics, Referral Pathways, and Technology Readiness for Home Modification

Participants from the 3 OAHMP grantees reported that they primarily served older adults aged 60 years and older, referred by local agencies or partner organizations, or self-referred. Assessments were typically led by occupational therapists, CAPS, or trained occupational therapy students. Notably, participants mentioned that home assessments sometimes involved the use of software, such as Home for Life and REDCap (Vanderbilt University), as well as informal observation or discussion [24,25].

Timelines from assessment to completed modifications varied among grantees, influenced by factors such as funding availability, staffing, contractor schedules, and program structure. Most participants indicated that their projects could be delayed beyond the initial estimates, even if assessments were completed promptly.

So you guys at any given time are probably working on 3 to 5 different projects at the same time. So again, sometimes it’s just a capacity issue in terms of who is next in line, who has all their paperwork in here, and then which site team is available to go do that whole (renovation).

[Org2]

Regarding the major applications for home modifications, agreed upon by participants from various grantee programs, traditional home modifications—such as grab bars, bathroom safety features, and entry ramps—were still identified as the core service to enhance clients’ safety, mobility, and accessibility. This continued reliance on traditional modifications, alongside the limited initial integration of smart technologies, reflects a broader pattern of cautious technology readiness among both clients and grantee organizations—shaped by clients’ age profiles, varying levels of digital familiarity, and the constraints of existing program structures.

These are things that our staff member can accomplish. So they are comfortable. High toilets, grab bars, handrails, tub cuts, solar powered security lights, levered door handles, levered faucets.

[Org2]

### Theme 2: Current Integration of Smart Home Technologies and Perceived Benefits

Several participants representing their OAHMP grantee programs reported that they had incorporated smart home devices into their modification projects, observed smart home technologies used by homeowners, and sometimes collaborated with contractors during installation. In addition, staff from one organization cited their indefinite delivery/indefinite quantity contract as a mechanism for formally recommending smart home devices.

The Amazon Echo, the Echo Show, the Echo dot, the Ring doorbell, and then the smart plugs and we also have on our IDIQ, which is what we use...for a lot of you know, items that we recommend a lot of the time. Some of the stuff on there does also have some smart home technology that can be recommended specifically by the occupational therapist, because they don’t typically look at the volunteer services as often as the CAPS do so they do have that option to add it in there if they feel a contractor should put it in.

[Org1]

But we have had lots of requests for smart technology from homeowners. And so that’s I’m glad I’m working with and listening to you all, because that’s something that we’re hearing more and more of; smart technology. And we need to figure out how to incorporate that better in our programs.

[Org3]

Most installations included Amazon Echo devices, Ring doorbells and cameras, smart plugs, and motion-activated lights. This pattern of selective adoption suggests that grantees prioritized devices that were low cost, easy to install, and directly addressed safety and emergency response needs, reflecting a pragmatic, needs-driven approach to technology integration within the constraints of existing program structures. These devices were valued for enhancing security, facilitating emergency response (eg, voice-activated 911 calls after a fall), and supporting independent living. Interestingly, 1 participant suggested additional applications of smart home technologies, such as detecting pets and wildlife and monitoring air quality for clients in rural or hazard-prone areas, which were not generally considered goals of incorporating those technologies into the programs.

One of our cases was really having 8 cats, and like raccoons like, you know, not even knowing that they’re up in the top, because our older adults aren’t traveling up to their second floor any longer. And so I don’t know just thinking creatively to toss it out there...Is there any smart tech to help with that? Particularly when, you know, even if they are animals that are pets, there’s that like environmental hazard for the home as well.

[Org2]

### Theme 3: Barriers to Adoption and Use of Smart Home Technologies

As OAHMP grantees, participants identified multiple barriers that limited the uptake and sustained use of smart home technologies by older adults. These included older adults’ digital literacy and confidence, proficiency in using smartphones and devices, concerns about privacy and data security, and the burden of device maintenance.

Do people have the wherewithal to even know what that is, and how it functions, and how to use it? Yes, some people do not have smartphones. They got the old flip phone, or they have their landlines. And okay, we would do this as a modification for them. But is it really gonna work for them?

[Org3]

I feel like we need to consider that you know all of us like, what’s the maintenance plan and where’s the education for when they need to replace that battery? So it doesn’t just become something that you know is now a trip hazard, you know, and potentially isn’t actually helping them, but could potentially hurt them, since it’s not functioning as intended. So there is maintenance associated with this.

[Org2]

Additionally, contextual factors frequently hinder the adoption of smart home technologies. Older adults living in rural areas often face unreliable internet connectivity and limited geographical access to certain services, which restricts the use of specific devices.

From where I’m based. It’s very rural, and our bandwidth is sometimes shoddy at best, so it’s very, um, sometimes that can be kind of a huge obstacle to be able to incorporate the smart home technology.

[Org1]

### Theme 4: Adoption and Implementation Strategies

For programs that have introduced smart home technologies, participants from those programs stated that current strategies to improve adoption include providing hands-on technical assistance—often through partnerships with university occupational therapy programs—and developing easy-to-use technology manuals for older adults. Interestingly, participants noted that their partnerships with local universities were viewed as mutually beneficial: older adults received guidance and support during the adoption process, while students gained hands-on learning experiences in aging in place and assistive technology.

They are a little bit reluctant because they are older adults and they don’t typically like to learn about the technology. But that’s kind of why we have the students go in because they go over it with them like very detailed, set up the accounts for them and everything, so that they don’t feel overwhelmed. And they actually also leave them a number that they can call whenever they have questions regarding any of the apps that they use.

[Org1]

Participants also recommended choosing devices that require minimal maintenance and can operate with limited internet connectivity.

I feel like we need to consider that you know all of us like, what’s the maintenance plan and where’s the education for when they need to replace that battery? So it doesn’t just become something that you know is now a trip hazard, potentially and isn’t actually helping them, but could potentially hurt them, since it’s not functioning as intended. So there is maintenance associated with this.

[Org2]

On the other hand, participants emphasized that the timing of adopting smart home technologies should be flexible—either during the initial assessment, as part of a standard package, or as optional add-ons later in the home modification process—to address older adults’ specific needs. Some older adults initially declined certain devices but became more receptive as their needs evolved, suggesting the value of a progressive adoption approach.

We do find that many times any type of recommendation that is made, if declined, is then requested again once a fall or something terrible, unfortunately, happens. So I mean… we do currently implement our smart home technology at each different touch point.

[Org1]

### Theme 5: Organizational Capacity and Change Readiness

All participants from various OAHMP grantee organizations agreed that integrating smart home technologies into home modification programs required organizational adaptation. Unlike client-facing adoption strategies, this organizational adaptation focused on building internal capacity—equipping staff, contractors, and volunteers with the knowledge and resources needed to deliver and sustain technology-enhanced services. This included training staff and contractors on device installation and troubleshooting, adding technical support roles, and fostering leadership buy-in. External partnerships with technology vendors, universities, and aging service providers were also seen as essential.

We are excited to increase the number of smart home tech devices that we’re able to offer. But along with that you know, we would hope that we can coordinate more intensive trainings for both ourselves as staff and our CAPS, but also like to train the trainers, so that we can adequately train our volunteers. Specifically our student volunteers to be able to train the older adults and their caregivers to be able to use the devices appropriately and not cause them more stress and frustration in trying to use the devices that you know we’re providing them.

[Org1]

Our volunteers perform these modifications. I think we may need some training for the volunteers, just what these smart technologies are, and how they can integrate those in the modifications, and how they can help a homeowner essentially set them up and make them work. So it would be some training that we would need for both ourselves and our and our volunteers who are doing this work.

[Org3]

Policy-wise, 1 participant emphasized that addressing funding limitations, staffing shortages, and technical expertise gaps was crucial for the sustainable implementation of the initiative at scale.

And then, you know, we’re nonprofit, so more funding to not only support the devices, because, as (one participant) mentioned, they are costly, but also the operations and capacity to roll out and maintain the program, so that we’re able to provide that staff support and training opportunities for the volunteers.

[Org1]

## Discussion

### Principal Results and Policy Implications

Findings from this focus group revealed that, while certain devices—particularly those supporting safety and emergency response—have begun to be integrated into home modification services for older adults, the broader implementation of smart home–equipped modifications remained limited. Barriers emerged across three levels: (1) older adults’ perceptions and digital readiness, (2) physical and contextual environmental constraints, and (3) programmatic factors, including policies and budget limitations that shape service delivery.

With an aging population, a lack of accessible, age-friendly US housing stock, and a need for cost-effective solutions to support aging in place, demand for home modifications is likely to increase in the coming years. Incorporating smart home technology into evidence-based home modification programs, such as CAPABLE and OAHMP, can provide a relatively low-cost and high-impact pathway to enhance opportunities for aging in place. Pairing smart home technology with home modifications can provide a variety of benefits, including (1) enhancing mobility, communication, social engagement, safety, independence, and health, (2) compensating for functional limitations, (3) reducing caregiver burden, and (4) preventing injurious falls. Implementing smart-home-enhanced modifications within existing evidence-based home modification programs, such as OAHMP, that support low-income older adults and those in underserved communities can broaden access to impactful, cost-effective services that promote aging in place.

The 3-level barrier structure identified in this study—individual, contextual, and organizational—is consistent with findings from the smart home adoption literature. Digital literacy limitations, privacy concerns, and the burden of device maintenance among older adults have been consistently reported in prior studies [16]. However, our findings extend this literature in 2 important aspects. First, rural internet unreliability emerged as a particularly salient contextual barrier in home modification programs, where clients are often low-income and geographically isolated—a population underrepresented in existing smart home adoption research. Second, organizational-level barriers, including staff training gaps, funding constraints, and leadership buy-in, represent a distinct and underexplored dimension of implementation challenges that go beyond individual user acceptance [26]. These findings suggest that addressing smart home technology adoption in home modification programs requires a multilevel implementation strategy rather than interventions focused solely on end user behavior. Emerging pilot programs are demonstrating the feasibility of such integrated approaches for low-income aging populations [27].

The implementation strategies identified by participants—including university partnerships with reciprocal learning benefits, progressive technology introduction across care touchpoints, and hands-on training—align with emerging evidence on effective support for technology adoption among older adults. Programs pairing students with low-income older adults in community settings have demonstrated strong potential to reduce the digital divide through a reciprocal model in which older adults receive personalized guidance while students gain practical experience. Similarly, introducing technology progressively at multiple touchpoints across the care continuum [28]—rather than as a one-time intervention—aligns with best practices in technology acceptance frameworks that emphasize meeting users at their current readiness level [20-23,29]. These findings reinforce the value of embedding adoption support within existing program structures rather than treating technology training as a separate intervention.

### Smart Home Technology Selection Criteria

To guide the selection of commercially available smart home devices for integration into existing home modification services, we derived practical criteria from participant responses during the focus group, organized thematically through the analytical process, and further developed these criteria based on established usability frameworks, including the System Usability Scale [30]. These criteria are organized across 5 perspectives: installation, usability, accessibility, sustainability, and security and privacy (Table 3).

Devices should be easy to install so they do not impose unnecessary burdens on contractors or disrupt home environments. Usability is essential; older adults require straightforward onboarding and intuitive operation without repeated training. Technologies should directly improve safety, mobility, or accessibility rather than complicate daily living. Notably, accessibility matters—interfaces must be clear, with indicators that accommodate sensory needs, and vendor technical support should be available to reduce reliance on program staff. Sustainability is critical for program feasibility, favoring devices that are durable, low maintenance, and free of ongoing costs. Finally, devices must safeguard both physical and digital safety by minimizing injury risks and ensuring strong data privacy protections.

**Table 3.** Criteria for selecting smart home devices.

Perspective	Criteria
Installation	Easy to install with minimum home modifications
Usability	Ease of onboarding Ease of use Easy adoption No memory interaction Improves safety, accessibility, or mobility
Accessibility	Interface design Clear indicators Technical support or customer service
Sustainability	Reasonable cost Low maintenance requirements No subscription fees
Security and privacy	Avoid risks of injury (eg, strangulation and asphyxiation) Avoid risks of injury (hazardous surfaces) Protect private information

To our knowledge, standardized criteria for selecting commercially available smart home devices specifically for integration into home modification programs have not been previously proposed in the literature. Prior systematic reviews have called for clearer classification standards and technology selection guidance to align device functions with user needs in aging in place contexts [18,31]. The 5-domain framework proposed here represents an initial step toward filling this gap and warrants further validation through broader stakeholder input and empirical testing.

### Limitations

This study has several limitations. First, only 3 OAHMP subawardee organizations participated, limiting the breadth of perspectives and potentially underrepresenting the diversity of all grantees across the program. Second, because participating organizations may differ in size, geographic location, or client characteristics from other OAHMP awardees, the views expressed may not be representative of the program as a whole. Furthermore, detailed organizational characteristics could not be reported at the organizational level to protect the confidentiality of participating organizations; however, purposive sampling ensured representation across rural, suburban, and national program contexts, as described in the Methods section.

From a design perspective, using a single 1.5-hour focus group session may have limited the depth of discussion and introduced dynamics such as unequal participation or social desirability bias. The analysis used a deductive coding approach based on the discussion agenda, which ensured alignment with study aims but may have limited the emergence of unanticipated or novel themes. Additionally, focus group participants represented a variety of roles within each home modification program. It is possible that the professional background of participants (eg, occupational therapist vs grant administrator) influenced perceptions of facilitators and barriers.

### Conclusions

Overall, this study underscores the potential of smart home technologies to complement traditional home modifications and enhance aging in place outcomes. Clear technology selection criteria to inform the development of a standard smart home technology package were developed. Barriers to the sustainable implementation of this initiative include older adults’ digital literacy, privacy concerns, and organizational capacity. Policy support and resource allocation will be crucial to increasing adoption rates, reducing inequities, and enhancing the impact of federally funded home modification programs. Future research should focus on validating the proposed selection criteria through broader stakeholder input, curating evidence-based device packages for integration into home modification programs, and evaluating the implementation and outcomes of smart home–enhanced modifications across diverse aging populations.

## Supplementary material

10.2196/89729 Multimedia Appendix 1 Focus group discussion guide used in the qualitative focus group study with Older Adults Home Modification Program grantee organizations.

## Abbreviations

CAPABLE: Community Aging in Place–Advancing Better Living for Elders
CAPS: Certified Aging-in-Place Specialist
OAHMP: Older Adults Home Modification Program
