# Supplementary material for: Smart Home Technology Integration in Home Modification Programs Serving Older Adults: Focus Group Study With Program Grantees
Source: JMIR Aging. 2026 Jul 16;9:e89729. doi: 10.2196/89729 (PMC13375085; doi:10.2196/89729)
Supplement: Multimedia Appendix 1 [file aging-v9-e89729-s001.docx]

# Multimedia Appendix 1. Focus Group Discussion Guide

The following questions were used to guide the focus group discussion. Questions were organized into three thematic sections aligned with the study aims. Probes were used at the facilitator's discretion to elicit further detail.

## Section A: Home Assessment and Modifications

### Clients and Referrals

1. Who is your primary target population for your home modification program?

2. How do you get your referrals in your organization? Are referrals internal, coming from healthcare providers, community agencies, or other sources?

### Current Home Assessment Process

3. Do you follow a standard assessment protocol or checklist?

4. Who typically conducts the assessment (e.g., occupational therapists, contractors, program staff)?

5. What is the balance between self-reported needs, direct observation, and performance-based assessments (e.g., watching clients perform tasks)?

6. Does the assessment typically include a walkthrough of the entire home or focus only on select areas?

### Time and Workflow

7. On average, how long does it take from the initial assessment to completing the home modifications?

*Probe: What factors contribute to longer or shorter timelines?*

### Common Modifications

8. What are the most common home modifications you currently provide?

*Probe: Are any of these driven by specific funding limitations or client priorities?*

### Use of Technology in Modifications

9. Do you currently incorporate any smart home or assistive technology in your modifications?

*Probe: (If yes) What types of technologies are being used (e.g., fall detection, smart lighting, voice-activated systems)?*

*Probe: (If yes) Who decides what technology is installed — the client/caregiver, the assessor, or another party?*

*Probe: (If yes) Do staff or contractors involved in the program receive training on smart or assistive technologies?*

*Probe: (If yes) How receptive are older adults and caregivers to smart technologies in their homes?*

*Probe: (If no) What are the main reasons or barriers? (e.g., cost, training, compatibility with funding requirements)*

## Section B: Smart Home Technology — Barriers and Facilitators

10. What benefits do you see in integrating or expanding the use of smart home technology in your current home modification services? For example, could it help older adults stay independent longer, prevent injuries, or provide peace of mind to caregivers?

*Probe: Could smart technology reduce your team’s workload or improve service delivery?*

11. What challenges or concerns would you expect in adding or expanding smart home technologies in your home modification work? For example: resistance from homeowners, concerns about digital literacy, data privacy, installation complexity, or ongoing maintenance.

*Probe: Can you think of any solutions to address these challenges?*

## Section C: Implementing Smart Technology into Your Organization’s Home Modification Program

12. How do you envision your organization integrating smart home technology into your current home modification services? For example, would it be part of the initial assessment, part of the standard modification package, or offered as an add-on for eligible clients?

13. In what ways do you think incorporating smart technology might influence your daily practice or your agency’s overall operations?

*Probe: Could this change how you assess homes, plan services, coordinate with contractors, or follow up with clients?*

14. What specific preparations or changes would your organization need to make to effectively incorporate smart technology into your services?

*Probe: Would this require staff training, hiring new expertise, or restructuring parts of your workflow?*

*Probe: How important would leadership engagement or organizational buy-in be to moving forward with this idea?*

*Probe: Would partnerships (e.g., with technology providers, aging services, or local universities) play a role?*

## Closing

15. Are there any other thoughts you would like to share on how to best implement smart technology in your practice setting?
